# Letter from Joseph A. Peters, Assistant Surgeon Twenty-First Regiment, New York State Volunteers

**Published:** 1861-09

**Authors:** Joseph A. Peters

**Affiliations:** Fort Runyon


ART. IV.—*Letter from Joseph A. Peters, Assistant Surgeon Twenty-First Regiment, New York State Volunteers.*

FORT RUNYON, July 30th, 1861.

MY DEAR DOCTOR :—Number one of your journal found its way into camp last night, and you may be sure was heartily welcomed. It has long been one of those facts, which the newspapers say, "no sane man can doubt," that the Medical Profession of Buffalo should have a journal, wherein to record its doings and observations, and in this enterprise you may be sure of our co-operation and support. Whatever of scientific interest we may find in our perigrinations into "Dixie," shall be faithfully transmitted to you as our contribution to the feast of reason, which discussions among medical men are sure to produce. I send you herewith a copy of the report on the vaccination of our regiment which we have just sent to the Surgeon General of the State of New York. I have added, you will see, some deductions from the figures furnished, not with the idea of proving anything, but merely to give every one who has a theory in regard to the operation of vaccine virus on the human system, a chance to prove it forthwith. I hereby give any one free leave to use said figures in any way they please, as *my* theory will not be published until I have written my great work on Pathology, which will be about the time some artists finish their great historical pictures.

There are two things of interest to the medical observer, which are to be seen in army practice ; one is the wounds and injuries incident to a battle, and the other is the effect of atmospheric conditions and personal habits on bodies of men. Of the first we saw an example on the 22d inst., although we were not in the battle, since our fort, forming the key to the Long Bridge, was soon filled with fugitives.

Eight thousand rations were issued here during the day, and our hospital facilities were taxed to their utmost to attend the wounded who walked and were brought in here. Dr. Treat was here and rendered prompt assistance, so that no one had occasion to go away uncared for. Many of the wounds having been temporarily dressed on the field, the day before required fresh dressing, and of course very few serious cases reached here, our records showing no more important operations than the extracting some bullets, and amputating some fingers, and toes. The hospitals at Washington do not contain many more cases of severe wounds proportionately to their numbers, than fell under our observation. It is perhaps that the grave cases were mostly left in the temporary hospitals at Centreville and Bull's Run, yet I am informed that there were not many cases of injuries requiring capital operations, even there. If these facts are authentic, they show that the enemy had troops as raw as our own, it being characteristic of such troops that their fire is too high, to effect much injury.

I think the bayonet was very little used by them, for we have seen but very few cases of bayonet wounds, and these few were mainly the result of accidents among our own men. Much has been said about their superiority in point of arms, but the wounds brought under our notice were made with the same kind of projectiles we use, viz : round bullets and buckshot. Of the progress and after treatment of the wounds I cannot give you any statement, as they were all transferred to the General Hospital in Washington and Georgetown. It is probable, however, that suppuration will generally take place, both from the fatigued and enfeebled condition of the men, and the imperfect ventilation inseperable from the warmth of the season and the character of the buildings which were not built with reference to hospital purposes. I am inclined to believe that large tents properly pitched would be much the best hospitals for such cases. We use for our sick no other hospital than such a tent, and it is surprising how readily the few cases it has been necessary to treat in hospital, have yielded to remedial measures.

I am just preparing our monthly report of sick and wounded for July, which is to be rendered the first of August, and a few facts in regard to our sanitary condition as a regiment, may not be uninteresting. We have not had a single death among us from sickness since our organization, and very little serious illness. I find on the books for July, 102 cases of acute diarrhœa, and 24 of dysentery ; of cholera morbus we report 17 cases ; of intermittent fever 17 ; remittent fever 1, and of a fever which has no precise nosological place, but which is palpably malarial, 8 cases. These last cases have all yielded readily to the use of a full dose of pulv. ruei. or pulv. jalapæ and hyd. chlor. mit. followed by full doses of quinine. The cases already detailed, with a few cases of bronchitis and tonsillitis, constitute the chief part of the disease we had to encounter during the month, so I believe we can safely return thanks for good health so far.

Our Fort is on the bank of the Potomac, which is just here a large marsh, and within its walls is considerable newly made earth and embankments, what effect this will have on our men during the coming months of August and September, remains to be seen.

My knowledge of military operations hereabouts being about as authentic as that possessed by the reporters, I refer you to their accounts for information on that point.

These jottings of a leisure moment, whether interesting to you or not will show my good will, and with best wishes for your success, I remain,

Yours faithfully,

JOSEPH A. PETERS.

[The Report on Vaccination referred to in this letter will be given in the October number.]

